# Improvement in cognitive and psychosocial functioning and self image among adolescent inpatient suicide attempters

**DOI:** 10.1186/1471-244X-6-58

**Published:** 2006-12-29

**Authors:** Ulla Hintikka, Mauri Marttunen, Mirjami Pelkonen, Eila Laukkanen, Heimo Viinamäki, Johannes Lehtonen

**Affiliations:** 1 University of Kuopio and Kuopio University Hospital, Department of Psychiatry, P.O. Box 1777, FIN-70211 Kuopio, Finland; 2 National Public Health Institute, Department of Mental Health and Alcohol Research, Helsinki, Finland; Hospital District of the University of Helsinki, Peijas Hospital, Department of Adolescent Psychiatry, Vantaa, Finland

## Abstract

**Background:**

Psychiatric treatment of suicidal youths is often difficult and non-compliance in treatment is a significant problem. This prospective study compared characteristics and changes in cognitive functioning, self image and psychosocial functioning among 13 to 18 year-old adolescent psychiatric inpatients with suicide attempts (n = 16) and with no suicidality (n = 39)

**Methods:**

The two-group pre-post test prospective study design included assessments by a psychiatrist, a psychologist and medical staff members as well as self-rated measures. DSM-III-R diagnoses were assigned using the SCID and thereafter transformed to DSM-IV diagnoses. Staff members assessed psychosocial functioning using the Global Assessment Scale (GAS). Cognitive performance was assessed using the Wechsler Adult Intelligence Scale, while the Offer Self-Image Questionnaire (OSIQ) was used to assess the subjects' self-image. ANCOVA with repeated measures was used to test changes from entry to discharge among the suicide attempters and non suicidal patients. Logistic regression modeling was used to assess variables associated with an improvement of 10 points or more in the GAS score.

**Results:**

Among suicide attempter patients, psychosocial functioning, cognitive performance and both the psychological self and body-image improved during treatment and their treatment compliance and outcome were as good as that of the non-suicidal patients. Suicidal ideation and hopelessness declined, and psychosocial functioning improved. Changes in verbal cognitive performance were more pronounced among the suicide attempters. Having an improved body-image associated with a higher probability of improvement in psychosocial functioning while higher GAS score at entry was associated with lower probability of functional improvement in both patient groups.

**Conclusion:**

These findings illustrate that a multimodal treatment program seems to improve psychosocial functioning and self-image among severely disordered suicidal adolescent inpatients. There were no changes in familial relationships, possibly indicating a need for more intensive family interventions when treating suicidal youths. Multimodal inpatient treatment including an individual therapeutic relationship seems recommendable for severely impaired psychiatric inpatients tailored to the suicidal adolescent's needs.

## Background

The prevalence of suicide attempts [1] completions [2] and mental disorders [3,4] increases in adolescence compared with childhood. Suicide is a major cause of death among adolescents in most western countries [5]. A previous suicide attempt is among the most significant factors for future suicidal behavior [6] and completed suicide [7,8]. A family history of suicide, low income, family adversity, parental divorce, and psychiatric disorders also increase the risk of suicidal behavior [8]. In suicide prevention, adolescent suicide attempters, especially those suffering from depression and other psychiatric disorders, are one particular high-risk group [2,6].

Suicidal behavior is common among adolescent inpatients. The reported rate of suicide attempts among adolescent inpatients has varied from 17% to 55% [9-11], and that of suicidal ideation from 33% to 57% [10,11]. Adolescents with previous suicide attempts and those with repeated attempts more commonly have affective and substance use disorders than non-suicidal youths and display externalizing behaviors and psychic distress as well as depressive symptoms and anger [9,12-14]. Moreover, they often have a long-term history of suicidal behavior associated with poor parent-child communication [6]. Referral to psychiatric inpatient treatment is recommended if an adolescent makes a suicide attempt with high lethality or if she/he has previously attempted suicide, has suicidal relatives or lacks family support [15].

Psychiatric treatment of suicidal youths is often difficult and non-compliance in treatment is a significant problem [16]. In a study of adolescent inpatients undergoing long-term treatment, negative feelings toward the body, poor protection of the body and body aberration were factors that differentiated suicidal and non suicidal inpatient groups [17]. Cross-sectional studies have reported risk factors for adolescent suicide attempts to include hopelessness and neuroticism [18]. Personality factors such as cognitive functioning and self-image are important elements of coping strategies and also in treatment interventions [19]. Follow-up reports have noted that distress symptoms, poor cognitive functioning, low self-esteem, and problems in interactions with family members associate with a poor treatment outcome in adolescent suicide attempters [20-23]. However, a more detailed characterization of cognitive functioning and aspects of self-image among adolescent suicide attempters, research on their long-term inpatient treatment and predictors of outcome are needed in order to develop effective interventions.

This study addressed the following questions: 1) How does psychosocial functioning, cognitive performance and self-image change during inpatient psychiatric treatment of adolescents with and without suicide attempts? 2) Are there clinical factors that associate with improvement in psychosocial functioning during inpatient treatment?

## Methods

### Subjects

A total of 88 youths aged 13 to 18 years, 53 girls and 35 boys, were consecutively referred from 1 March 1997 to 31 December 1999 for psychiatric treatment to the adolescent psychiatric inpatient unit of Kuopio University Hospital, Finland. Inclusion criteria in the present study were that the adolescent was: 1) between 13 and 18 years of age, 2) not referred only for brief intervention in response to a temporary crisis (less than four weeks), 3) able to co-operate, and 4) suffered from a clinically significant psychiatric disorder. Adolescents exhibiting primarily substance abuse or dependence and those who needed only a brief intervention were referred to another treatment setting. Sixty-five adolescents met the inclusion criteria, 1 female and 2 males declined to participate [24]. All patients and their parents provided written informed consent before entering the study. The study was approved by the Ethics Committee of Kuopio University Hospital and the University of Kuopio.

At study entry, during a semi-structured interview, a psychiatrist screened the adolescents for suicide attempts before the inpatient treatment (no/yes/when). We excluded inpatients with psychotic disorders (n = 7). The sample (n = 55) was then divided into those who had attempted suicide during the previous 12 months (suicide attempters; n = 16) and those with no previous suicide attempts (non-suicidal patients; n = 39).

### Treatment

The treatment was part of an existing clinical service and the assessment of relationship and outcome variables were included in the service. Treatment schedules were individualized from a range of psychosocial interventions, and included individual psychotherapy, psychotropic medication when appropriate and sport, occupational, and art group therapy. Each adolescent received individual psychotherapy, two 45-minute sessions weekly, conducted by the case manager nurse, who was a member of a multidisciplinary team. The nurses had been trained in a two-year course on psychodynamic psychotherapy as a part of multidisciplinary treatment and psychotherapy was conducted under supervision. Case manager nurses also interacted with other patients in the ward but did not have regular sessions with them. Family sessions were conducted once every three weeks, mostly led by a social worker. The treatment included specialized hospital school. The adolescents spent every second weekend at home. Adolescents' treatment schedules were focused and evaluated individually at entry, after two weeks and then once every six weeks by the multidisciplinary team. To determine the appropriate time for discharge from hospital the medical staff team assessed psychosocial functioning, suicidal, and the safety of the foster environment. They also discussed with the adolescent and her/his parents whether it was safe to enter outpatient treatment. The decision to discharge the patient was made by a psychiatrist after these assessments.

### Study procedure

The two-group pre-post test prospective study design included assessments by a psychiatrist, a psychologist and medical staff members as well as self-rated measures collected at entry and on discharge. The medical staff of the hospital ward was trained to perform all procedures and assessments in a standardized manner for research purposes. DSM-III-R diagnoses were assigned using the SCID [25], and thereafter transformed to DSM-IV diagnoses by checking each DSM-IV diagnostic criterion from the SCID-interview, patient files and other data collected for the study. Multiple psychiatric diagnoses were allowed. General IQ was 70 or more in all participants, and the participants had not been diagnosed as having any neurological diseases.

Data on parental basic socio-demographic and socio-economic backgrounds included: 1) marital status of the biological parents (married, divorced, separated (each: no/yes)), 2) family on living allowances, 3) mental health problems among family members and 4) death of the mother or father (no/yes). Data on family characteristics were collected during semi-structured interviews with the parents conducted by staff members. The patients' academic achievement, including behavioral problems (many/some or none), were assessed at entry together with hopelessness (one item yes/no) and alcohol and drug misuse in a semi-structured interview by a psychiatrist. Data on indirect self-destructive behavior defined as repeated exposure to life-threatening danger without suicidal intent and having been bullied at school during the previous six months were collected at entry. Prior psychiatric treatment and the time interval between suicide attempts and referral to inpatient treatment were assessed. Anhedonia, current depressive mood, and suicidal ideation were assessed using the questions of the SCID I interview. Medical staff members recorded the number of treatment interventions daily (therapy sessions with the individual case manager nurse, assessments conducted by a psychiatrist, a psychologist, a social worker and an occupational therapist, family sessions, treatment meetings of the staff-team, group therapy sessions and number of school days).

A team of 6–8 staff members assessed the psychosocial functioning of the patients at entry and on discharge using the Global Assessment Scale (GAS) [26]. The adolescents' cognitive performance (FIQ) was assessed by a clinical psychologist using five sub-tests of the Wechsler Adult Intelligence Scale – Revised, adapted for use in the Finnish population [27]. The verbal intellectual level (VIQ) was assessed using three scales (Similarities, Vocabulary and Digit Span) and nonverbal performance intelligence (PIQ) was assessed using two scales (Digit Symbol and Block Design). Standard points were adjusted to correspond to the IQ [27]. The overall sum of standard points was the combined sum. The sum of standard points (Similarities, Vocabulary, and Digit Span) was multiplied by 6/3, while the sum of standard points (Digit Symbol and Block Design) was multiplied by 5/2. To be consistent, we converted the raw scores from sub-tests to scaled score equivalents using the corresponding norms for the ages of 16–17 years. The subscales of Similarities, Digit Span, Digit Symbols and Block Design that were used in this study are suitable for emotionally disturbed patients, and their scaled scores do not differ from those of the Wechsler Intelligence Scale for Children [28,29]. A Logical Prose Subtest, Story recall A, from the Wechsler Memory Scale WMS; [30] was used as a measure of immediate and delayed logical verbal memory. The first score was based on the units immediately recalled. The delayed score was based on the units recalled from the story 60 minutes later. The List Learning Test (LLT) was used to assess learning and recall [31]. The participants learned and recalled aloud 15 semantically unrelated words in four consecutive trials, using version A at entry and B after six months or on discharge. The word lists were used alternatively, eliminating the effect of learning.

The Offer Self-Image Questionnaire (OSIQ) for adolescents was used to assess the self-images of the patients [32-34]. The OSIQ has been validated among Finnish adolescents [35,36]. In this study, Psychological Self (impulse control, emotional tone, body image), Social Self (social relations, vocational and educational goals) and Familial Self (family relationships) were assessed. A higher score in the OSIQ indicates better functioning.

### Data analysis

The student's t-test, chi-squared test, Fisher's exact test and exact Mann-Whitney U-test were used to compare suicide attempters and non-suicidal subjects. Paired samples t-test or exact Wilcoxon signed ranks test were used to analyze differences between entry and discharge and repeated measures analyses of variance with covariates (suicide attempt or not, current depressive mood, anhedonia at entry, number of days attending school, number of individual therapy sessions, psychotropic medication on discharge) was used to analyze changes in cognitive performance and self image. All tests were applied on a two-tailed basis. Normality and homoscedasticity were visually checked.

Univariate logistic regression analyses were applied to define variables to be included in the final multivariate model associated with the dependent variable (improvement 10 points or more in GAS score). All the explanatory variables were first entered into a univariate model as single predictors (age, gender, patient in the suicidal group, number of therapy sessions, duration of inpatient treatment, GAS score at entry, an improved body-image, number of team sessions, MDD diagnosis (no/yes), number of group sessions, number of Family sessions, Verbal cognitive performance score on discharge, medication on discharge, Psychological Self, Social Self and Familial Self). Being classified as a suicide attempter or not and the predictors showing statistical significance of less than 0.2 in the univariate analysis were included in the multivariate stepwise logistic regression model. Backward selection procedure was used in order to find the final reduced model. Logistic regressions were used also when considering whether suicide attempt history related to categorical clinical variables (suicidal ideation, indirect self-destructive behaviour, depressive mood, anhedonia) adjusted for baseline suicidal ideation. The results are expressed as ORs (odds ratios) with their 95% confidence intervals. In interpreting the results of the final model, the level of significance was set at 0.05. Statistical analyses were performed with the statistical package SPSS for Windows 14.0, and post hoc sample size estimation was carried out with the nQuery Advisor^® ^4.0 program.

## Results

### Clinical characteristics at entry

The proportion of girls was 88% (14/16) in the suicide attempter group, and 59% (23/39) among the non-attempters. Suicide attempts had taken place during the previous 12 months (median 27 days). Eight (50%) of these patients had attempted suicide during the previous month. In the suicide attempter group, there were seven adolescents with one, two with two, and one with three or more suicide attempts in the prior year. No one of the non-attempters had attempted suicide prior to the 12-month timeframe. The mean age of the suicide attempters was 15.4 years (SD 0.8, range 14–17 years) and that of the non-attempters 15.1 years (SD 1.0, range 13–17 years; p = ns). The principal psychiatric diagnoses (DSM-IV) in the groups (suicide attempters/non-attempters) were major depressive disorder (n = 13/15, z = 2.88, p = 0.004), dysthymia (n = 0/3, z = 1.14, p = ns.), bipolar disorder (n = 2/6, z = 0.29, p = ns.), conduct disorder (n = 1/10, z = 1,62, p = ns.), eating disorders (n = 0/3, z = 1.14, p = ns.) and anxiety disorders (n = 0/2, z = 0.92, p = ns.). Eleven (69%) of the suicide attempters and 23 (59%) of the non-attempters had comorbid psychiatric disorders (z = 0.69, p = ns.).

Suicidal ideation, hopelessness, current depressive mood, indirect self-destructive behavior, and alcohol and drug misuse were more common among the suicide attempters than non-attempters (Table 1). There was no statistically significant between-group difference in psychosocial functioning at entry (mean GAS score ± SD: suicide attempters 41.8 ± 8.3 versus non-attempters 39.3 ± 8.7, p = ns.). Fourteen (87.5%) suicide attempters and 32 (82.1%) non-attempters had previously utilized psychiatric services. A family history of being on living allowances was more common among suicide attempters than among non-attempters (Table 1).

**Table 1:** Comparison of sociodemographic and clinical characteristics between suicidal and non-suicidal adolescent inpatients at entry

	Adolescents		
Groups:	Suicidal (n = 16)	Non-Suicidal (n = 39)	

	N (%)	N (%)	P-value^1^

Parents divorced	8 (50.0)	19 (48.7)	n.s.
Family on living allowances	7 (43.8)	5 (14.7)	0.032
Mental health problems in family	5 (31.3)	10 (26.3)	n.s.
Father or mother dead	1 (6.3)	3 (7.7)	n.s.
Academic achievement declined	14 (87.5)	41 (89.1)	n.s.
Hopelessness	15 (93.8)	22 (56.4)	0.006
Alcohol and drug misuse	7 (43.8)	4 (10.5)	0.010
Indirect self-destructive behavior	14 (87.5)	13 (34.2)	<0.0005
Bullied at school	10 (62.5)	19 (50.0)	n.s.
Current daily depressive mood^a^	10 (62.5)	13 (33.3)	0.046
Anhedonia^a^	10 (62.5)	14 (35.9)	0.066
Suicidal ideation^a^	7 (58.3)	7 (24,0)	0.048

n.s. = non significant, ^1^Fisher's Exact Test; ^a ^Based on SCID interview;

Except for use of psychotropic medication during treatment, no statistically significant differences were found in treatment variables between adolescents with and without suicide attempts (Table 2). There was not statistically significant difference in the mean duration of inpatient treatment between suicide attempter and non-attempter adolescents (M = 175 days, SD = 99 vs. M = 145 days, SD = 81, respectively). Non-verbal cognitive performance scores at entry were better among the suicide attempters than among the non-attempters (Table 3; PIQ; p = 0.040). The Psychological Self score (p = 0.003), including the emotional tone (p = 0.003) and body-image sub-scale scores (p = 0.001), were significantly poorer among the suicide attempters compared with the non-attempters at entry (Table 4).

**Table 2:** Comparison of treatment variables between suicidal and non-suicidal adolescent inpatients

	Adolescents		
Groups:	Suicidal (n = 16)	Non-Suicidal (n = 39)	

	Mean (SD)	Mean (SD)	P-value

Number of individual therapy sessions	52 (15.8)	32 (19.0)	n.s.
Number of psychiatric assessments	19 (17.6)	13 (15.8)	n.s.
Number of psychologist assessments	9 (1.7)	8 (3.4)	n.s.
Number of social worker assessments ^b^	5 (3.0)	5 (5.0)	n.s.
Number of occupational therapist assessments ^b^	21 (9.2)	10 (7.6)	n.s.
Number of family sessions	6 (3.5)	6 (4.1)	n.s.
Number of team sessions	9 (11.6)	6 (4.8)	n.s.
Number of group therapy sessions ^b^	16 (14.5)	13 (12.5)	n.s.
Number of days attending school	72 (40.7)	62 (44.0)	n.s.
Psychotropic medication during treatment	12 (75.0%)	14 (35.9%)	0.016

n.s. = non significant; T-Test; ^b^Mann-Whitney U-test

**Table 3:** Cognitive performance and memory functioning at entry and on discharge in suicide attempters and non-attempters adolescent inpatients. Comparison at entry and on discharge and change between groups

	Adolescent					
Groups	Suicide attempters (n = 16)^a,b^		Non-Attempters (n = 39)^a,b^		Statistical significance of the change between the groups (n = 55)^1^	

Cognitive performance and memory functioning	At entry Mean(SD)	On discharge Mean(SD)	At entry Mean(SD)	On discharge Mean(SD)	Subjects Effects: *F(Suicide attempt or not)	Main Effect: *F(Suicide attempt or not) and F(Number of therapy sessions)

General cognition (WAIS-R/FIQ) ^c^	99.7 (10.7)	107.9 (9.6)***	95.7 (10.7)	101.7(11.5)***	n.s.	n.s.
-Verbal cognition (WAIS-R/VIQ)	97.0 (12.1)	104.0 (10.2)***	95.2 (10.7)	98.2 (10.4)**	*F(1,36) = 4.99, p = 0.032	n.s.
-digit span	7.7 (2.1)	8.5 (2.1)*	7.7 (2.6)	8.5 (2.9)**	n.s.	n.s.
-vocabulary	8.2 (2.7)	9.3 (2.4)*	7.5 (2.6)	7.8 (2.6)	*F(1,36) = 7.54, p = 0.009	F(1,36) = 4.72, p = 0.036
-similarities	8.3 (3.8)	10.7 (3.2)**	7.8 (3.3)	8.7 (3.2)**	n.s.	n.s.
-Nonverbal cognition (WAIS-R/PIQ)	103.5 (9.8)	112.1 (10.8)***	97.9 (11.3)	106.1(15.3)***	n.s.	n.s.
-block design	9.7 (3.5)	11.7 (3.5)**	9.0 (2.9)	10.7 (3.3)***	n.s.	n.s.
-digit symbol	9.7 (2.3)	11.5 (1.9)***	8.8 (2.4)	10.6 (3.0)***	n.s.	n.s.
Immediate logical memory (WMS)	6.9 (3.2)	9.1 (3.5)	7.1 (4.8)	8.1 (4.5)	F(1,36) = 3.86, p = 0.057	n.s.
Delayed logical memory (WMS)	4.6 (3.2)	6.4 (3.0)	5.0 (4.6)	5.7 (4.0)	n.s.	n.s.
Learning memory (LLT/total)	33.1 (6.3)	35.1 (8.7)	33.4 (8.8)	36.1 (9.6)**	n.s.	n.s.
Delayed recall memory (LLT)	7.5 (2.8)	8.0 (2.8)	8.6 (3.3)	8.7 (3.4)	n.s.	n.s.
Delayed recognition memory (LLT)	13.7 (1.2)	13.4 (1.5)	13.5 (1.8)	14.2 (2.7)	n.s.	n.s.
Delayed memory (delayed recall/learning memory total × 100)	22.2 (5.1)	23.5 (6.0)	23.7 (7.1)	22.6 (7.3)	n.s.	n.s.

^a ^Paired sample T-test, ^b ^Wilcoxon Signed Ranks non-parametric test; ^1 ^adjusted means; *p < 0.05, **p < 0.01, ***p < 0.001, n.s. = non significant, ^1 ^Comparisons are adjusted for following covariates: suicide attempt or not, current depressive mood, anhedonia at entry, number of days attending school, number of individual therapy sessions, psychotropic medication on discharge)

**Table 4:** OSIQ scores at entry and on discharge in suicide attempters and non-attempters adolescent inpatients and comparison between groups

	Adolescents					
Groups:	Suicide attempters (n = 16)^a^		Non-Attempters (n = 39)^a^		Statistical significance of the change between groups ^1 ^(n = 55)	

	At entry Mean (SD)	On discharge Mean (SD)	At entry Mean (SD)	On discharge Mean (SD)	Subjects Effects: * F(Suicide attempt or not)	Main Effect: * F(Suicide attempt or not) and F(Number of therapy sessions)

Psychological Self	98.1 (19.7)	118.6 (23.8)**	115.2 (26.1)	125.5 (19.1)**	n.s.	F(1,35) = 3.93, p = 0.055
Impulse control	32.0 (7.4)	38.3 (7.4)**	36.0 (7.5)	38.1 (6.6)*	n.s.	n.s.
Emotional tone	37.0 (7.7)	45.9 (10.0)***	45.3 (7.4)	48.4 (7.7)**	n.s.	n.s.
Body-image	30.2 (7.0)	35.6 (7.7)**	37.1 (7.0)	39.0 (6.9)*	n.s.	F(1,36) = 5.82, P = 0.021
Social Self	81.5 (7.1)	85.2 (10.7)	85.2 (13.9)	89.6 (8.5)**	n.s.	n.s.
Social relationships	37.3 (5.2)	39.3 (6.5)	40.6 (7.5)	42.3 (5.1)	n.s.	n.s.
Vocational and educational goals	44.2 (3.8)	45.9 (6.0)	44.6 (8.5)	47.3 (5.7)	n.s.	n.s.
Familial Self	74.4 (15.0)	78.6 (12.3)	79.3 (16.2)	86.8 (14.3)*	n.s.	F(1,29) = 6.56, p = 0.016

^a ^Paired sample T-test, ^1 ^adjusted means; n.s. = non significant; *p < 0.05, **p < 0.01, ***p < 0.001

^1 ^Comparisons are adjusted for following covariates: suicide attempt or not, current depressive mood, anhedonia at entry, number of days attending school, number of individual therapy sessions, psychotropic medication on discharge)

### Changes in psychosocial functioning and clinical variables during treatment

There was a statistically significant improvement in mean (± SD) GAS scores from admission to discharge in both groups: among suicide attempters from 41.8 (8.3) to 52.1 (9.1) (p = 0.003) and among those with no suicide attempts from 39.1 (9.7) to 51.2 (8.1) (p = 0.001). GAS scores did not differ between the groups on discharge. Two patients in the suicide attempter group (12.5%) and one (2.6%) in the non-attempter group attempted suicide during the treatment. All the attempters were females. The timing of the suicide attempts was close to the end of treatment among two subjects. The third patient made several suicide attempts during the treatment that lasted for five months. All the three patients received individual psychotherapy and they had family sessions. One of the attempters had bipolar I disorder, her medication consisted of mirtazapine and a mood stabilizer. The other attempter received citalopram and thioridatzine, while the third attempter received only anxiolytic medication. Seven (43.8%) suicide attempters and 7 (17.9%) non-attempters received SSRI medication (p = 0.046). Five (31.3%) attempters and 2 (5.1%) non-attempters received antidepressants remeron from the NASSA-group (p = 0.008). One (6.3%) attempter and 3 (7.7%) non-attempters received mood stabilizer (p = ns.). Six (37.5%) attempters and 8 (20.5%) received antipsychotic medication (p = ns.).

When adjusted for baseline suicidal ideation the rate of positive change in suicidal ideation was 55.6% among patients with suicide attempts and 21.1% among non-attempters with no statistically significant difference between the two groups (OR = 0.62, 95% CI 0.40–9.65). The rate of positive change in indirect self-destructive behavior was 46.7% among suicide attempters and 21.6% among non-attempters (OR = 2.6, 95%CI 0.50–13.7) and in hopelessness 80.0% among suicide attempters and 51.1% among non-attempters (OR = 6.28, 95% CI 0.61–64.9). Depressive mood declined in 35.7% among suicide attempters and in 33.3% of non-attempters (OR = 0.87, 95% CI 0.18–4.08), and anhedonia in 28.6% among the attempters and in 31.3% of the non-attempters, respectively (OR = 0.43, 95% CI 0.01–2.30).

### Changes in cognitive performance during treatment

In unadjusted comparisons general cognitive performance (FIQ), as well as verbal (VIQ) and nonverbal cognitive performance (PIQ), improved during treatment in both patient groups (Table 3). On discharge, suicide attempters performed tentatively better in the similarities subscale of verbal cognition than the non-suicidal patients (Table 3; p = 0.060). IQ was at the common average levels [27] both at entry and on discharge in both patient groups. Non-attempters' improved learning memory functioning during treatment.

In adjusted comparisons, the improvement in verbal cognitive performance, and in the vocabulary subscale, was more pronounced among the suicide attempters (Table 3, column 'Subjects Effects'). More severe depressive mood associated with less improvement in general cognition [F (1, 36) = 4.14, p = 0.049] and non-verbal cognition [F (1,36) = 7.26, p = 0.011] including the block design subscale [F (1, 36) = 6.95, p = 0.012] as well as the digit symbol subscale [F (1, 36) = 7.92, p = 0.008]. Use of medication associated with improvement in the non-verbal cognitive performance [F (1, 36) = 4.11, p = 0.050]. General cognition [F (1, 36) = 6.64, p = 0.014], and in non-verbal cognitive performance [F (1, 36) = 6.24, p = 0.017] and the digit symbol subscale [F (1, 36) = 4.15, p = 0.049] changed in both groups during treatment and the change were similar in the patients groups. The number of therapy sessions associated with improvement in immediate logical memory (Table 3). The higher the number of therapy sessions (Table 3 column "Main Effect") and the number of days attending school the better was improvement in the vocabulary subscale during treatment [F (1, 36) = 5.62, p = 0.023].

### Changes in self image during treatment

In unadjusted comparisons the total OSIQ scores in the Psychological Self, including impulse control, emotional tone and body image, improved during treatment in both patient groups. (Table 4). Among subjects with no suicide attempts, also Social self and Familial Self scores improved. In adjusted comparisons there were no between-group differences (Table 4, column "Subjects Effects"). The high number of therapy sessions associated with improvement in Psychological Self, the Body image subscale and Familial Self (Table 4, column "Main Effect"). More severe depressive mood tended to associate with less improvement in the Psychological Self [F (1, 36) = 4.02, p = 0.053] including improvement in the impulse control subscale [F (1, 36) = 5.06, p = 0.031]. The more severe anhedonia the lower was the change in the social relationships subscale [F (1, 36) = 4.85, p = 0.034].

### Factors associated with improvement in psychosocial functioning

Ten patients (63%) in the suicide attempter group improved in their GAS score by 10 points or more compared with 22 (56%) in the non-attempter group. The predictors showing statistical significance of less than 0.2 in the univariate analysis (number of therapy sessions, duration of inpatient treatment, GAS score at entry, an improved body-image and number of team sessions) and being classified as a suicide attempter or not were entered into the multivariate stepwise logistic regression model. Backward selection was used in order to find the final reduced model.

The final multivariate logistic regression model explained 26,2% of the variance in the change in psychosocial functioning (GAS score) during treatment (Table 5). Having an improved body-image associated significantly with a higher probability of improvement in psychosocial functioning while higher GAS score at entry was associated with lower probability of functional improvement.

**Table 5:** Final solution of the logistic regression analysis: factors associated with improvement in psychosocial functioning (10 points or more in GAS score) among adolescents (n = 55) during psychiatric inpatient treatment

Backward Stepwise (Wald)	Variables	Beta	Odds Ratio	95% Confidence Interval	P-value
Step 4	Patient in the suicidal group	1.458	4.30	0.82–22.43	0.084
	GAS score at entry (continuous)	-0.095	0.91	0.84–0.99	0.030
	Body-image score on discharge (continuous)	0.103	1.09	1.00–1.22	0.044

n.s. = non significant df = (1, 55)

## Discussion

Over one quarter of adolescent inpatients in this study had attempted suicide during the previous 12 months. Fifty per cent of these attempters were suicidal at time of admission. Most of them had major depression, and substance abuse, hopelessness, suicidal ideation, and indirect suicidal behavior were common. Although the suicide attempters were severely impaired in their Psychological self, their improvement in verbal cognitive performance and vocabulary were more pronounced than non-attempters during treatment. Their body- and self image, and psychosocial functioning improved and suicidal ideation and hopelessness declined during treatment. Their treatment compliance was as good as that of the non-suicidal inpatients.

The proportion of adolescent inpatients who had attempted suicide in our sample (26%) falls within the range previously reported (17–55%) [9-11]. Our finding that suicide attempts were significantly more common among female than male adolescent inpatients also replicates previous findings [9,11]. In accordance with previous research on sex, living allowances and morbidity [6,8,14,37-39], multiple distress symptoms and economic hardship in the family associated with suicidal in our sample. Consistently with previous research we found a strong association between suicide attempts, depression and substance abuse [40], and hopelessness [1]. Inpatients with suicide attempts also had more commonly indirect self-destructive behaviour and poorer attitudes and feelings towards the body and body protection than those in the non-suicidal group, which is consistent with previous studies on suicidal adolescents [9,17,41].

Our findings suggest that the IQ profile may be lowered among suicide attempters due to the burden of clinical symptoms and associated psychosocial factors. This is consistent with previous studies reporting suicidal behavior to be associated with poor social and problem-solving skills and other cognitive deficits [42-44]. The results of the present study are also in line with the finding from a previous five-year follow-up study that a positive outcome associated with a high IQ [45].

Positive changes were recorded in attitudes towards the self-image (Psychological Self) with respect to reported bodily (Body Image) experiences, but no changes were found in attitudes towards parental treatment and bonding (Familial Self) among adolescents who had attempted suicide. Suicidal adolescents may have had a low tactile sensitivity and responsiveness and a low emotional investment in the body because of mental pain influenced by internal and external sources such as loss [46], wounding in earlier family life and poor interaction with the parents [47].

Although the adolescent inpatients in this study were emotionally deeply wounded and had a poor body and familial self, recovery in psychosocial functioning was associated with positive changes in the body and self image. One explanation for the poor recovery in familial relationships may be that the clinical interventions were insufficient to counteract problems in parental and family life or that a longer time is needed for this type of recovery. It seems that a sufficiently long comprehensive psychiatric inpatient treatment including individual psychotherapy and family sessions may be an effective treatment modality for suicide attempter adolescents with severe psychiatric problems [2,6]. Hopelessness, suicidal ideation and depressive mood appear to be critical components in the treatment of suicidal adolescents [21,48], and significant indicators of future suicidality [1]. Particular attention should be paid to relieving hopelessness and depressive mood in the treatment of adolescent suicide attempters.

Despite the prospective design, the regionally representative consecutively-referred inpatient sample, comprehensive data collection, use of well-validated measures, and structured diagnostic interviews, the results of the present study need to be viewed in the context of several limitations. Firstly, due to the small sample size the risk of false negative findings (type II statistical errors) cannot be ruled out. Several statistical tests were performed, which may increase probability of type I error. Secondly, there were only two ratings of cognitive and psychosocial functioning, and Psychological, Social and Familial Self-Images during the treatment precluding assessment of change in different phases of the treatment. Thirdly, the use of a control group would have been desirable in a study assessing clinical outcomes [49]. One cannot leave severely disordered adolescents untreated or offer them less intensive care. It has recently been suggested that well-designed naturalistic studies can yield results that are as valuable as randomized controlled trials [50].

## Conclusion

The findings of the present study illustrate the importance of a thorough assessment of adolescent suicide attempters and that a multimodal treatment program with an individual therapeutic relationship seems to improve their psychosocial functioning and self-image. The treatment schedule should include models of enrichment and rehabilitation of cognitive and self-image functioning, and family sessions.

## Competing interests

The author(s) declare that they have no competing interests.

## Authors' contributions

UH planned the study, organized and participated in the data collection, performed the statistical analyses and drafted the manuscript. MM and MP participated in interpretation of the analyses, in drafting the manuscript. EL, HV and JL participated in the design and coordination of the study and helped to draft the manuscript. All authors read and approved the final manuscript.

## Pre-publication history

The pre-publication history for this paper can be accessed here:


